# Using hydroxyl radical footprinting to explore the free energy landscape of protein folding

**DOI:** 10.1016/j.ymeth.2015.02.018

**Published:** 2015-11-01

**Authors:** Antonio N. Calabrese, James R. Ault, Sheena E. Radford, Alison E. Ashcroft

**Affiliations:** School of Molecular and Cellular Biology, Astbury Centre for Structural Molecular Biology, University of Leeds, Leeds LS2 9JT, UK

**Keywords:** Hydroxyl radical footprinting, Fast photochemical oxidation of proteins, Protein structure, Protein folding, Electrospray ionisation-mass spectrometry, Liquid chromatography–mass spectrometry

## Abstract

Characterisation of the conformational states adopted during protein folding, including globally unfolded/disordered structures and partially folded intermediate species, is vital to gain fundamental insights into how a protein folds. In this work we employ fast photochemical oxidation of proteins (FPOP) to map the structural changes that occur in the folding of the four-helical bacterial immunity protein, Im7. Oxidative footprinting coupled with mass spectrometry (MS) is used to probe changes in the solvent accessibility of amino acid side-chains concurrent with the folding process, by quantifying the degree of oxidation experienced by the wild-type protein relative to a kinetically trapped, three-helical folding intermediate and an unfolded variant that lacks secondary structure. Analysis of the unfolded variant by FPOP–MS shows oxidative modifications consistent with the species adopting a solution conformation with a high degree of solvent accessibility. The folding intermediate, by contrast, experiences increased levels of oxidation relative to the wild-type, native protein only in regions destabilised by the amino acid substitutions introduced. The results demonstrate the utility of FPOP–MS to characterise protein variants in different conformational states and to provide insights into protein folding mechanisms that are complementary to measurements such as hydrogen/deuterium exchange labelling and *Φ*-value analysis.

## Highlights

• Fast photochemical oxidation of proteins discriminates between protein conformers.
• Global increases in the extent of oxidation correlate with protein unfolding.
• Localisation of oxidation to the peptide/amino acid level provides further insights.

## 1 Introduction

Mass spectrometry (MS)-based techniques are being employed increasingly to assist in the structural characterisation of biomolecules, such as proteins, protein assemblies and protein–ligand complexes [1], [2]. Numerous methods have been developed for this purpose, including covalent labelling, hydrogen/deuterium exchange (HDX), and chemical crosslinking, which can be mapped at a residue-specific level using MS analysis [1], [2], [3], [4], [5], [6].

Fast photochemical oxidation of proteins (FPOP) is an emerging MS-based technique with the potential to elucidate conformational changes in proteins resulting from folding/unfolding processes [7], [8], [9], [10], [11], [12], [13], [14], [15] and for probing changes in solvent accessibility that accompany ligand binding [16], [17], [18]. The FPOP approach requires the addition of small quantities of H_2_O_2_ (typically 0.02% (v/v)) to protein-containing solutions immediately prior to infusion through a capillary system and irradiation with a pulsed laser [19]. This procedure generates hydroxyl radicals that react with potentially any solvent accessible amino acid side-chain (although reaction rates for each amino acid differ significantly) [4], resulting typically in +16 Da mass additions, although additional modifications may also be observed [4], [20]. Addition of quenching reagents such as glutamine or histidine reduces the hydroxyl radical lifetime to approximately 1 μs [17], [12], [21]. The labelling time after each laser pulse, therefore, is faster than most protein unfolding/refolding reactions, allowing each protein conformation to be labelled effectively instantaneously in an experiment. Hence, both spatial (amino acid labelling sites) and temporal information regarding protein folding can be obtained [7], [8], [9], [10], [11], [12], [13], [14], [15]. FPOP–MS serves as a complementary technique to the more established method of HDX-MS [22], but the covalent nature of the modifications in FPOP–MS permits more rigorous sample handling procedures downstream of the labelling process without loss of information.

**Fig. 1:**
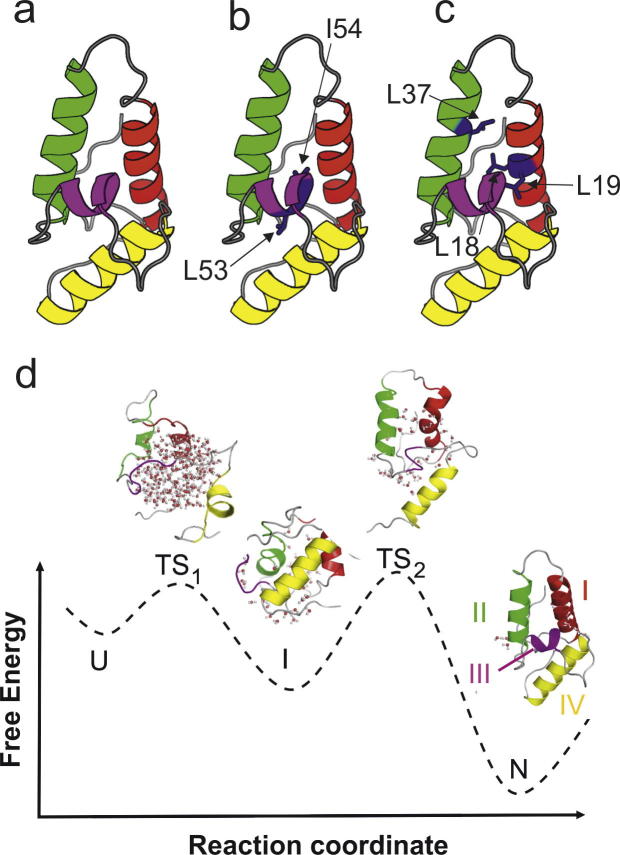
(a) The native structure of Im7, which has a four-helix fold (PDB accession 1AYI [41]). The locations of the amino acid substitutions with Ala are shown for (b) DM Im7 and (c) TM Im7. (d) Schematic of the folding energy landscape of Im7 showing the species populated during folding (U, unfolded state; I, intermediate state; N, native state; TS_1_ and TS_2_, the first and second transition states). Structures generated from molecular dynamics simulations are shown for TS_1_, I and TS_2_[27], [38]. Water molecules are shown (red spheres) that are displaced from the core as the protein folds. The four helices present in native, wild-type (WT) Im7 are coloured (I) red, (II) green, (III) purple, and (IV) yellow.

Mutational analysis of protein folding and unfolding kinetics (*Φ*-value analysis) is one of the most powerful approaches used to dissect protein folding mechanisms [23]. The information from these analyses can then be used to introduce mutations that stabilise partially folded intermediates or more highly unfolded states of a protein at equilibrium, allowing their direct structural analysis. Here, we have chosen to study the four-helix, bacterial immunity protein, Im7 (Fig. 1) [24] and two mutant constructs. The first construct, L53A I54A Im7 (DM Im7), has two amino acid changes in helix III [25]. This species populates the on-pathway folding intermediate at equilibrium, and has been shown previously to retain native-like secondary structure in helices I and IV, with partial formation of helix II and complete loss of helix III [25], [26], [27]. The second variant, L18A L19A L37A Im7 (TM Im7), was employed because these amino acid substitutions prevent Im7 folding and stabilise the unfolded state at equilibrium. This unfolded variant has been shown to be compact, retaining few fixed tertiary interactions and lacking persistent helical structure [28].

Here, we use MS-based techniques to interrogate the structures of Im7, DM Im7 and TM Im7. Initial studies utilising native electrospray ionisation-ion mobility spectrometry–mass spectrometry (ESI-IMS–MS) failed to distinguish between native Im7, the intermediate DM Im7 and unfolded TM Im7. We show that when the proteins were labelled using the FPOP technique, a correlation between the extent of structural destabilisation and the degree of covalent modification was observed. This demonstrates that FPOP can be implemented successfully to probe differences in the conformational properties and dynamics of proteins. Furthermore, localisation of oxidation sites shows that destabilisation of secondary structure in a particular region leads to an increase in the levels of oxidative labelling in the specific region of the sequence involved. This is consistent with FPOP permitting discrimination of the conformational dynamics of specific amino acid side-chains in regions of protein structure, producing data that are complementary to information about the main chain provided by HDX.

## 2 Materials and methods

### 2.1 Materials

Im7 constructs containing an N-terminal hexahistidine tag (ME(His)_6_) were overexpressed in *Escherichia coli* and purified as described previously [24], [29].

### 2.2 Circular dichroism

Far-UV circular dichroism (CD) spectra were recorded on a Chirascan CD spectrophotometer (Applied Photophysics, Leatherhead, Surrey, UK) using a 1 mm path length cuvette. Solutions contained protein at a concentration of 0.1 mg/mL in 10 mM sodium phosphate buffer, pH 7.0. Spectra shown are the average of three scans that were acquired over the range 190–260 nm with a bandwidth of 1 nm and a scan speed of 20 nm min^−1^. The buffer contribution was subtracted from the spectrum of each sample.

### 2.3 Native electrospray-ion mobility spectrometry–mass spectrometry

Lyophilised proteins (5 μM) were dissolved in 50 mM ammonium acetate buffer, pH 6.9 and desalted using Micro BioSpin 6 (Bio-Rad, Hemel Hempstead, UK) columns. ESI-IMS–MS experiments were performed using a Synapt HDMS mass spectrometer (Waters Ltd., Wilmslow, Manchester, UK). Sample introduction was achieved by nano-ESI using in-house prepared gold-plated borosilicate capillaries. Typically, a capillary voltage of 1.4 kV was applied, the cone voltage was set to 40 V, and a backing pressure of 4.5 mBar was used. IMS separation was achieved using a wave height of 5 V, and a wave velocity of 250 ms^−1^. Drift times were calibrated using experimentally determined CCSs of native proteins and applying a procedure described in detail elsewhere [30], [31], [32], [33]. CCSs were calculated from coordinates deposited in the Protein Data Bank using a scaled projection approximation (PSA) [34]. Aqueous CsI was used for *m*/*z* calibration. Data were processed using MassLynx v4.1 and Driftscope v2.5 software (Waters Ltd., Wilmslow, Manchester, UK).

### 2.4 Fast photochemical oxidation

Samples contained protein (10 μM) in 10 mM sodium phosphate buffer, pH 7.0 and were supplemented with 20 mM l-glutamine. 0.02% v/v H_2_O_2_ (final concentration) was added immediately prior to laser irradiation from a 30% v/v stock solution. The sample was infused through a fused silica capillary (inner diameter 100 μm), which had a window etched with a butane torch, at a flow rate of 20 μL/min. Hydroxyl radicals were generated by exposing the sample (through the etched window) to irradiation from a Compex 50 Pro KrF excimer laser operating at 248 nm (Coherent Inc., Ely, UK) with frequency of 15 Hz and a laser beam width of <3 mm at the point of irradiation. These solution, flow and laser pulse conditions ensure each bolus of protein-containing solution is exposed only once to laser irradiation and that conformational averaging during labelling does not occur, as the labelling reaction is on a faster time scale than any unfolding event which may occur, due to the presence of the quenching reagent l-glutamine [12], [19], [21]. The capillary outflow (100 μL) was collected in a 1.5 mL tube that contained 20 μL of a 100 mM l-methionine/1 μM catalase solution in 10 mM sodium phosphate buffer, pH 7.0 to degrade residual H_2_O_2_ and quench any hydroxyl radicals. Control samples were handled in the same fashion, without being subjected to laser irradiation, to correct for any background oxidation that may occur on the timescale of the FPOP experiment. 50 μL of protein-containing solutions were digested with trypsin for 16 h at 37 °C using a 1:50 (w/w) enzyme:protein ratio. The samples were frozen on dry ice and stored at −80 °C until analysis.

### 2.5 Liquid chromatography–mass spectrometry

Following proteolysis, proteins were analysed intact using an Ultimate 3000 nanoLC system (Dionex, ThermoFisher UK Ltd., Hemel Hempstead, UK). 1 μL of each sample was loaded onto a MassPREP protein desalting column (Waters Ltd., Manchester), that was washed with 2% (v/v) solvent B in solvent A (solvent A was 0.1% (v/v) formic acid in water, solvent B was 0.1% (v/v) formic acid in acetonitrile) for 5 min at 40 μL min^−1^. After valve switching, the bound proteins were eluted using a fast gradient of 2–40% solvent B in A over 1 min at 0.5 μL min^−1^. The column was subsequently washed with 95% solvent B in A for 6 min and re-equilibrated with 5% B in A for the next injection. The column eluant was directly infused into a Synapt G2S mass spectrometer (Waters Ltd, Wilmslow, Manchester, UK) via a Z-spray nanoflow electrospray source.

For mapping of peptides, data-dependent LC–MS/MS was conducted on an Ultimate 3000 nanoLC system (Dionex, ThermoFisher UK Ltd., Hemel Hempstead, UK) interfaced to a Synapt HDMS mass spectrometer (Waters Ltd., Wilmslow, Manchester, UK). Peptides (1 μL) were injected onto a PepMap C18, 100 μm i.d. × 15 cm column and then separated by gradient elution of 2–45% solvent B (0.1% (v/v) formic acid in acetonitrile) in solvent A (0.1% (v/v) formic acid in water) over 60 min at 0.35 μL min^−1^.

The Synapt HDMS was operated in positive TOF mode using a capillary voltage of 3.8 kV, cone voltage of 20 V, backing pressure of 2.47 mbar (8.57 for the Synapt G2S) and a trap bias of 4 V. The source temperature was 80 °C. Argon was used as the buffer gas at a pressure of 5.0 × 10^−4^ mBar (9.17 × 10^−3^ mBar for the Synapt G2S) in the trap and transfer regions. Mass calibration was performed by a separate injection of sodium iodide at a concentration of 2 μg/μl. For peptide LC–MS/MS, GluFib was infused as a lock mass calibrant with a one second lock spray scan taken every 30 s during acquisition. Ten scans were averaged to determine the lock mass correction factor. Data acquisition was achieved using data dependent analysis with a one second MS scan over an *m*/*z* range of 350–3000 being followed by three 1 s MS/MS scans taken of the three most intense ions in the MS spectrum over an *m*/*z* range of 50–2000. The collision energy applied was dependent upon the charge state and mass of the ion selected. Dynamic exclusion of 60 s was used. Data processing was performed using the MassLynx v4.1 suite of software supplied with the mass spectrometer and PEAKS Studio 7 (Bioinformatics Solutions, Ontario, Canada).

Peptide level quantitation of oxidation was achieved by calculating background-corrected % modified values using LC/MS signal intensities of the modified peptides (*I*_modified_) relative to their unmodified counterparts (*I*_unmodified_), using the following equation:

(1) $$\%\;\text{modified}=\frac{\sum I_{\text{modified}}}{I_{\text{unmodified}}+\sum I_{\text{modified}}}$$

Values were normalised to take into account background levels of oxidation by performing the same analysis on samples not subjected to laser irradiation. The data for DM Im7 and TM Im7 peptides (% modified_mutant_) were then normalised using the % modified values for the corresponding peptides in native Im7 (% modified_WT_) to determine the change in oxidation observed, using the following equation:

(2) $$\%\;\text{change}=\%\;{\text{modified}}_{\text{mutant}}-\%\;{\text{modified}}_{\text{WT}}$$

## 3 Results and discussion

### 3.1 Global changes in secondary structure are induced by mutations

For this study, WT Im7 and two variants were selected for analysis using FPOP because of their known differences in structure which have been described previously using a wealth of kinetic and structural analyses [25], [28]. DM Im7 populates an intermediate, partially folded state at equilibrium which contains three of the four native α-helices, whilst TM Im7 populates an unfolded ensemble which is compact but lacks persistent secondary structure [25], [28].

**Fig. 2:**
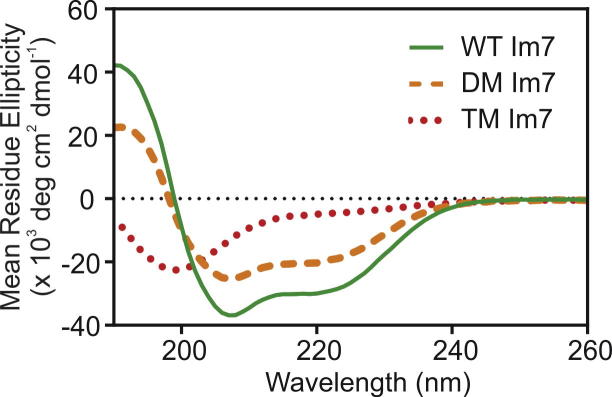
CD Spectroscopy of Im7 constructs. Native WT Im7 (green solid line) exhibits a CD spectrum characteristic of an α-helical protein. DM Im7 (orange dashed line) has reduced helical content, whilst TM Im7 (red dotted line) lacks the native helices.

The differences in the secondary structure content of each variant under the solution conditions used for oxidative labelling were studied by circular dichroism (CD) spectroscopy (Fig. 2). The resulting spectra show that native WT Im7 has a spectrum with negative maxima at 208 and 222 nm, characteristic of a protein with a high degree of α-helicity. The intensities of these negative maxima decreased in DM Im7, indicating a loss of secondary structure consistent with the unfolding of helix III (Fig. 2). Finally, TM Im7 exhibits a CD spectrum which is characteristic of a protein that is substantially unfolded (Fig. 2). Deconvolution of the observed CD spectra indicates that native WT Im7 has approximately 50% α-helical content, whilst DM Im7 is approximately 45% helical and for TM Im7 the helical content is decreased further to approximately 9%, consistent with previous results [25], [28].

### 3.2 The Im7 variants have similar collision cross-sections measured using ESI-IMS–MS

**Fig. 3:**
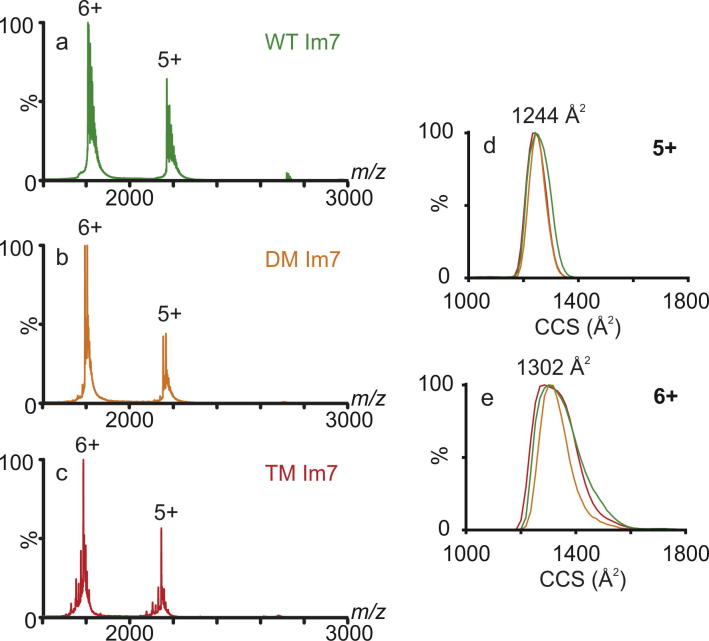
Native ESI-IMS–MS spectra of (a) Im7, (b) DM Im7, and (c) TM Im 7. (d and e) CCSs of the Im7 constructs, using the colour scheme in (a) to (c). Arrival Time Distributions (ATDs) of (d) 5+ and (e) 6+ charge states are shown. The theoretical CCS from the Im7 structure (PDB accession 1AYI [41] is 1165 Å^2^). The measured CCSs of the 5+ ions are within 7% of the estimated value from the crystal structure, indicating good agreement with the theoretical CCS value (the error associated with the calibration of CCSs is ⩽7.5%) [30].

We next set out to determine whether native ESI-IMS–MS could be used to detect differences in the structural properties of Im7, DM Im7 and TM Im7. Despite these proteins having very different secondary structure content, they all adopt relatively compact globular structures in solution (with hydrodynamic radii values for WT Im7, DM Im7 and TM Im7 measured as 19.3, 19.0 and 26.1 Å, respectively) [26], [28]. The native ESI-IMS–MS spectra of these proteins are qualitatively similar, displaying just two main charge states (5+ and 6+) consistent with compact structures (Fig. 3a–c). The rotationally averaged collision cross-sections (CCSs) of the ions at the two lowest observed charge states did not differ significantly for the different protein variants (1244 Å^2^ (5+) and 1302 Å^2^ (6+)), indicating that these gas-phase conformers are too similar in CCS to be resolved (Fig. 3d and e). Despite TM Im7 having a larger hydrodynamic radius in solution and presumably adopting a larger number of conformations compared with its partially folded and native counterparts, the CCS distributions of the proteins are indistinguishable (Fig. 3d and e). This is likely because the hydrophobic interactions which are stabilising the core of TM Im7 in solution are perturbed in the gas-phase leading to collapse [28], [35], [36]. Given that ESI-IMS–MS is particularly useful for determining the extent of disorder [37], i.e. conformational flexibility, in proteins, the data obtained here for Im7 likely reflect the global lack of disorder in the unfolded and intermediate states that is present in solution.

### 3.3 Global changes in oxidative labelling are observed in different conformational variants of Im7

**Fig. 4:**
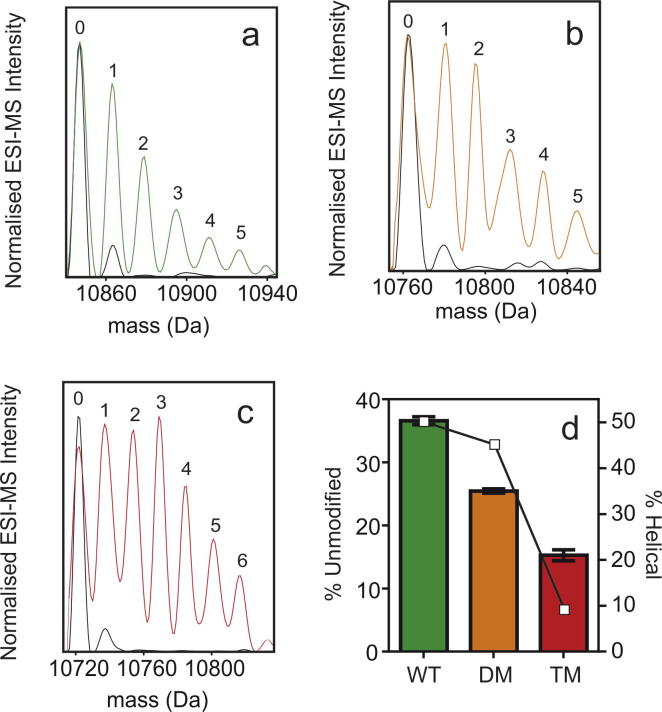
Deconvoluted mass distributions of (a) WT Im7, (b) DM Im7, and (c) TM Im7. Mass distribution of the H_2_O_2_ treated (but not laser irradiated) samples are shown in black, and mass distributions of the laser irradiated samples are shown in colour. Peaks corresponding to the protein precursors are labelled 0, with other peaks labelled according to the number of oxygen atoms incorporated as a result of oxidative labelling (1, 2, 3, etc). (d) Estimated % of unmodified protein remaining after FPOP (mean ± SEM of three replicates analysed in triplicate is shown as bars) and estimated % helical content as determined by CD spectroscopy (shown as white squares) [25], [28].

Im7, DM Im7 and TM Im7 were labelled using the FPOP procedure outlined in the Materials and Methods section. Intact samples of the labelled proteins were desalted online and analysed by MS. Fig. 4a–c show the deconvoluted mass distributions of the oxidised samples, with +16 Da mass additions observed to the corresponding precursor upon laser irradiation in the presence of H_2_O_2_.

Oxidation of amino acid side-chains by hydroxyl radicals is not only dependent on solvent accessibility, but also on amino acid identity [4]. Consequently, it is important to consider the amino acid substitutions present in the Im7 variants studied here and any influence these may have on the rate of the oxidation reaction, and hence the extent of modification observed. In this instance, all of the amino acid substitutions introduce an alanine residue at the expense of either a leucine or isoleucine. The rate constants for the reactions of these amino acids with hydroxyl radicals show that alanine reacts more slowly with hydroxyl radicals than either leucine or isoleucine, with rate constants of 7.7 × 10^7^, 1.7 × 10^9^ and 1.8 × 10^9^ M^−1^ s^−1^, respectively [4]. Thus, these substitutions reduce the susceptibility of the protein to oxidation, ruling out the possibility that any increased levels of oxidation simply result from alteration in the amino acid composition of the variants.

Analysis of the mass distributions of the oxidised intact proteins and quantification of the ratio of unoxidised to oxidised protein present in each sample was also performed. These data indicate that introduction of amino acid substitutions in the Im7 sequence results in the protein experiencing higher levels of oxidation (Fig. 4d) (i.e. there is less unmodified protein present after FPOP). Given that the variants remain compact, these differences most likely result from alterations in the average secondary structure content and average compactness (i.e. the width of the conformational ensemble) of each protein. In the case of DM Im7, both helix III and the C-terminal seven amino acids of helix II are destabilised [26], whilst for TM Im7, only 9% helical structure, on average, persists [28]. The measured percentages of unmodified protein remaining after FPOP are plotted in Fig. 4d, along with the estimated percentage of α-helical structure in each protein (from CD spectroscopy data). The results indicate that as the average α-helical content of the protein decreases (as does the thermodynamic stability [25], [27], [28]), the protein becomes more susceptible to oxidation. Thus, the data highlight the power of FPOP coupled with MS analysis as a probe of the conformational properties of proteins. The technique is highly sensitive to the presence of a hydrogen bonding network that would exclude water and thus prevent side chain oxidation by hydroxyl radicals in solution. This is analogous to HDX-MS, where backbone amide hydrogens are exchanged for deuterons, a process that is retarded when residues are involved in hydrogen bonding networks or are buried from solvent [22].

### 3.4 Local changes in oxidative labelling are observed for different Im7 variants

**Fig. 5:**
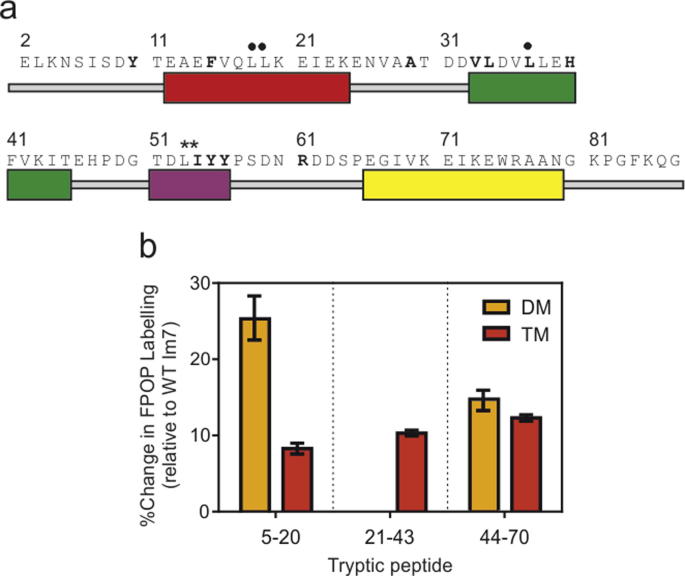
(a) Sequence and secondary structure of WT Im7. Numbering shown is for native Im7, the construct used contains an additional 8 residues at the N-terminus (MEHHHHHH-) for expression and purification purposes [24], [28], [29]. The four helices are coloured (I) red, (II) green, (III) purple, and (IV) yellow. The sites of mutations in DM Im7 are indicated with asterisks and the sites of mutations in TM Im7 with black circles. Residues where oxidation was identified in the three peptides investigated are shown in bold. (b) Peptide level quantification of oxidation levels in DM Im7 and TM Im7 (relative to the corresponding unoxidised peptide in the same sample). Data were normalised to the extent of modification observed in WT Im7 to track changes in the different proteins. Peptide 21–43 from DM Im7 shows no increase in modification extent relative to WT Im7.

The sites of oxidation were localised to the peptide level by use of LC–MS analysis of tryptic Im7 peptides (Fig. 5a). Quantification of the extent of oxidation was performed for the three tryptic peptides in which differences in oxidation levels between the three proteins were detected. The data were normalised to determine the change in oxidation experienced by each peptide from the protein variants relative to the corresponding peptide from WT Im7 (Fig. 5b). The results show that TM Im7 has an increase in oxidative labelling across the entire sequence compared with native, WT Im7 (Fig. 5b), consistent with the global changes in oxidation experienced by the protein (Fig. 4d). By contrast, DM Im7 exhibits spatial differences in the changes in oxidation level observed, with only two of the three peptides showing increases in oxidation levels compared with the WT Im7 (Fig. 5b). One of these peptides (44–70) corresponds to regions of the protein incorporating helix III, which is known to be unfolded in DM Im7 [27], [38]. The peptide of DM Im7 which does not show an increase in oxidation relative to WT Im7 (21–43) spans a portion of helix I and helix II, which are native-like or partially formed, respectively, in the intermediate state (Fig. 1d). Sequence coverage was poor beyond residue 70 of Im7 (due to the presence of many trypsin-cleavable residues), so this region of the protein was not analysed in sequence specific detail.

The oxidation sites were next localised to specific amino acid residues in the three tryptic peptides (Fig. 5a) by analysis of LC–MS/MS data. LC–MS/MS spectra were assigned using automated software and all assignments were verified manually. The rate constants for the reaction of amino acid side-chains with hydroxyl radicals are known, and span four orders of magnitude (from a rate of 1.7 × 10^7^ M^−1^ s^−1^ for Gly to 3.4 × 10^10^ M^−1^ s^−1^ for Cys [4], [5]). The most reactive residues, in order of reactivity when they are equally accessible in bulk solution, have been shown to be: Cys, Met > Trp, Phe, Tyr > Pro > His > Leu [39]. It can be seen that the side-chains most susceptible to oxidation are those which are sulphur-containing, followed by those which are aromatic. Consequently, these residues are considered the most reliable probes of solvent accessibility and are considered further here [5].

The peptide comprising the N-terminal unstructured region and helix I (residues 5–20) was found to be susceptible to oxidation at two residues. In WT Im7, the oxidation site in this peptide was identified solely as at position Y10 (Fig. 5a). A significant increase in the oxidation of this peptide in DM and TM Im7 indicates there is increased flexibility in the N-terminal region of the protein that is not observed in the native state (Fig. 5b, left). Additionally, residue F15, which is buried in the native state, was identified as an oxidation product only in DM and TM Im7, indicating that this residue becomes solvent accessible and available for modification only in the intermediate and unfolded variants. In a second peptide comprising part of helix I and all of helix II (residues 21–43), modification sites were identified for residue A29 in the loop between helix I and II, and in helix II itself (Fig. 5a). This helix is partially formed in DM Im7 and its C-terminal portion has been shown to be in dynamic exchange in the intermediate state using NMR spectroscopy [26]. However, there were no residues in this region where a significant increase in oxidation level was observed in the intermediate DM Im7 compared with WT native Im7 (Fig. 5b, centre), likely because the residues involved remain buried [26]. Conversely, unfolding and exposure of this helix in TM Im7 leads to a significant increase in the oxidation efficiency throughout this region (Fig. 5b, centre). Interestingly residue F41, which forms a part of the hydrophobic core of Im7, was not oxidised in any of the samples, despite Phe being one of the most reactive side chains and a reliable probe of solvent accessibility. This indicates that in all the Im7 variants residue F41 remains buried. Solution and molecular dynamics simulation data have shown this particular residue to be involved in many non-native contacts which stabilise the intermediate state, possibly explaining why this residue is not modified in DM Im7 despite its high reactivity [24], [38]. Likewise, aromatic clusters persist in TM Im7, also likely involving residue F41 [28]. The major oxidation sites identified in the peptide comprising helix III and part of helix IV (residues 44–70) were Y55 and Y56, which are both located in helix III, with residue Y55 being partially buried in the native-state while Y56 is more solvent exposed [40] (Fig. 5a). Thus, the susceptibility of these residues to oxidation is significantly enhanced upon unfolding of this helix in both DM and TM Im7 (Fig. 5b, right). Together, the results demonstrate that FPOP coupled to MS can provide insight into the structure, flexibility and dynamics of amino acid side-chains in different protein conformers, and does not suffer from the effects of conformational exchange that often rule out direct analyses of partially folded proteins using NMR spectroscopy.

## 4 Conclusions

We have demonstrated the ability of FPOP–MS to differentiate between three different sequence variants of Im7 which are similar in overall compaction but have significant differences in secondary structural content and persistent tertiary structural interactions. We show how information about side-chain accessibility in the different conformational states (native, partially folded and globally unfolded) obtained by use of FPOP coupled with MS analyses is complementary to the results from HDX which report on the structure and solvent accessibility of the main-chain, and also to direct measurements of dynamics made by using NMR spectroscopy. Moreover, the rapid timescale of FPOP avoids conformational averaging, which often precludes direct structural analyses of non-native states using NMR. FPOP thus adds to the arsenal of techniques now available to interrogate protein folding and dynamics, promising new information about the role of individual side-chains in determining the nature of protein folding energy landscapes.
